# Editorial: Implications of senescence in cancer immunology

**DOI:** 10.3389/fimmu.2026.1998525

**Published:** 2026-09-30

**Authors:** Alessandra Zingoni, Gordon Cook, Laura Senovilla

**Affiliations:** 1 Department of Molecular Medicine, Laboratory Affiliated to Istituto Pasteur-Fondazione Cenci Bolognetti, Sapienza University of Rome, Rome, Italy; 2 Cancer Research UK Clinical Trials Unit, Leeds Institute of Clinical Trial Research, University of Leeds, Leeds, United Kingdom; 3 Institute of Biomedicine and Molecular Genetics (IBGM), Higher Council for Scientific Research (CSIC) - University of Valladolid (UVA), Valladolid, Spain

**Keywords:** cancer immunology, senescence, senescence-associated secretory phenotype, senescent tumor microenvironment, therapy-induced senescence

Cellular senescence has emerged as a fundamental biological process at the intersection of cancer, immunity, and aging (1). Traditionally regarded as a durable cell-cycle arrest that protects tissues from malignant transformation, senescence is now recognized as a highly dynamic and context-dependent state. Senescent cells remain metabolically active and can profoundly remodel their environment through the senescence-associated secretory phenotype (SASP), with consequences that may range from tumor suppression and immune activation to chronic inflammation, immune evasion, and disease progression (2, 3). The contributions collected in this Research Topic, *“Implications of senescence in cancer immunology,”* highlight the complexity of this phenomenon and point toward an emerging paradigm in which senescence is not merely an endpoint of cellular damage, but a modifiable determinant of tumor–immune interactions.

As emphasipzed by Qian et al., the dual nature of cellular senescence represents a central challenge for cancer immunology. Senescence can restrain tumor growth by preventing proliferation of damaged cells and by generating signals that facilitate immune recognition and clearance. However, persistent senescent tumor cells may acquire properties that favor their survival and immune escape. The SASP can establish a chronic inflammatory and immunosuppressive microenvironment, while senescence-associated immune checkpoints may further compromise immune surveillance. Thus, whether senescence is beneficial or detrimental may depend on its depth, duration, cellular context, and ability to engage effective immune clearance. This conceptual framework provides a common thread linking the diverse contributions included in this Research Topic.

This complexity becomes particularly evident when senescence is considered in the context of aging. Gergues et al. emphasizes the reciprocal relationship between senescence and natural killer (NK) cells, whose ability to eliminate malignant and senescent cells may decline with age. Immunosenescence and inflammaging can therefore weaken a critical mechanism of senescent cell surveillance precisely when the burden of senescent cells increases. Wang et al. extends this concept to oncolytic virus therapy, highlighting the immune frailty of older patients as a potentially important determinant of both efficacy and tolerability. These observations underscore the need to move beyond chronological age and incorporate functional measures of immune competence and frailty when designing and evaluating cancer immunotherapies for older populations.

The therapeutic implications of therapy-induced senescence (TIS) represent another major thread of this Research Topic. Chemotherapy, radiotherapy, and targeted agents can induce a senescent phenotype in tumor cells, potentially creating an immunogenic state that can subsequently be exploited for tumor elimination. The “one-two punch” strategy (i.e., first inducing senescence and subsequently eliminating senescent cells) therefore offers an attractive framework for integrating senescence into precision oncology. However, senescence induction alone is unlikely to be sufficient (4). The work discussed by Menendez et al. highlights mitochondrial apoptotic priming as a potentially critical and underappreciated determinant of the response of TIS cells to CAR-T and CAR-NK immunosenolytic therapies. Importantly, the persistence of pre-existing anti-apoptotic dependencies after senescence induction suggests that the therapeutic vulnerability of senescent cells may be, at least in part, predictable. This provides a rationale for companion diagnostics such as BH3 profiling and for rational combinations of cellular immunotherapy with agents targeting apoptotic dependencies.

A complementary strategy is to interfere directly with mechanisms that allow senescent cancer cells to evade immune elimination. The review by Zhao et al. identifies CD47 as a molecular bridge between TIS and immune tolerance. By engaging the CD47–SIRPα axis, senescent tumor cells may inhibit phagocytic clearance and contribute to their persistence. Targeting this pathway could therefore transform a senescent state from a reservoir of therapy-resistant cells into a therapeutically exploitable vulnerability. More broadly, these observations reinforce the concept that the surfaceome of senescent cells may provide a valuable source of targets for immunosenolytic approaches.

The potential clinical relevance of senescence-associated molecular programs is further illustrated by the computational and multi-omics studies included in this collection. In bladder cancer, Du et al. developed a senescence-related signature associated with immunotherapy response, prognosis, and the immune landscape, identifying PPIL3 as a potential regulatory target. Similarly, the multi-omics analysis of hepatocellular carcinoma by Tuergan et al. identified an MCM7-associated senescence axis linked to tumor progression, immune dysfunction, and potential resistance to immunotherapy. Although such signatures require prospective and functional validation, they illustrate how senescence-related molecular features may eventually contribute to patient stratification and treatment selection.

Importantly, the implications of senescence extend beyond tumor growth and therapeutic response. Antonangeli et al. introduce the “innervated niche” as another dimension of the senescent tumor microenvironment, proposing that senescence-associated inflammation may contribute to neuroinflammation and cancer-associated pain. This perspective expands the biological consequences of tumor senescence and raises the intriguing possibility that immune-mediated targeting of senescent cells, including through NK cells, could simultaneously influence tumor control and cancer-associated symptoms.

Collectively, the eight contributions in this Research Topic support a shift from viewing senescence as a static tumor-suppressive state toward understanding it as a dynamic component of the tumor ecosystem. Several important questions remain open. How can beneficial, transient senescence be distinguished from persistent, tumor-promoting senescence? Which senescent-cell surface markers are sufficiently selective to permit safe immunosenolysis? How does the aging immune system alter the efficacy of senescence-targeting therapies? And can senescence-associated biomarkers reliably predict which tumors will benefit from senogenic–senolytic combinations?

Addressing these questions will require integration of functional senescence assays, single-cell and spatial approaches, immune profiling, and longitudinal clinical studies. Ultimately, the therapeutic opportunity may not lie simply in inducing or eliminating senescence, but in controlling its timing, cellular location, and interaction with the immune system. The studies gathered in this Research Topic provide important steps toward this goal and suggest that manipulating the dialogue between senescent cells and immune effectors may become an increasingly relevant strategy for precision cancer immunology.

## References

[1] Ajoolabady A Pratico D Bahijri S Tuomilehto J Uversky VN Ren J . Hallmarks and mechanisms of cellular senescence in aging and disease. Cell Death Discov. (2025) 11:364. doi: 10.1038/s41420-025-02655-x 40759632 PMC12322153

[2] Wiley CD Campisi J . The metabolic roots of senescence: mechanisms and opportunities for intervention. Nat Metab. (2021) 3:1290–301. doi: 10.1038/s42255-021-00483-8 34663974 PMC8889622

[3] Wang B Han J Elisseeff JH Demaria M . The senescence-associated secretory phenotype and its physiological and pathological implications. Nat Rev Mol Cell Biol. (2024) 25:958–78. doi: 10.1038/s41580-024-00727-x 38654098

[4] Prasanna PG Citrin DE Hildesheim J Ahmed MM Venkatachalam S Riscuta G . Therapy-induced senescence: opportunities to improve anticancer therapy. J Natl Cancer Inst. (2021) 113:1285–98. doi: 10.1093/jnci/djab064 33792717 PMC8486333

